# Correlation between small dense low-density lipoprotein cholesterol and carotid artery intima-media thickness in a healthy Chinese population

**DOI:** 10.1186/s12944-015-0143-x

**Published:** 2015-10-29

**Authors:** Hao Shen, Li Xu, Jingfen Lu, Tianbo Hao, Chunfang Ma, Honglin Yang, Zhaoyang Lu, Yongchun Gu, Tonghua Zhu, Guorong Shen

**Affiliations:** Department of Clinical Laboratory Medicine, The First People’s Hospital of Wujiang, Affliated Wujiang Hospital of Nantong University, Suzhou, China; Department of Ultrasonography, The First People’s Hospital of Wujiang, Affliated Wujiang Hospital of Nantong University, Suzhou, China; Department of General Surgery, The First People’s Hospital of Wujiang, Affliated Wujiang Hospital of Nantong University, Suzhou, China; Department of Central Laboratory, The First People’s Hospital of Wujiang, Affliated Wujiang Hospital of Nantong University, Suzhou, China

**Keywords:** Small dense low-density lipoprotein cholesterol, Carotid artery intima-media thickness, Chinese subjects

## Abstract

**Background:**

Small dense low-density lipoprotein cholesterol (sdLDL-C) concentration was useful in the assessment of the presence of cardiovascular diseases (CVD) and its severity. We examined whether SdLDL-C is more closely associated with carotid artery intima-media thickness (CA-IMT), a surrogate measure of atherosclerosis, than LDL-C and traditional CVD risk factors in Chinese healthy subjects.

**Methods:**

We measured CA-IMT, blood pressure (BP), sdLDL-C, glucose metabolism and lipid in 183 native Chinese healthy subjects. CA-IMT was assessed by ultrasonography, and sdLDL-C concentrations were measured by a homogenous assay. Pearson's correlation coefficient analyses and Multiple regression analyses were used to examine the relationships between CA-IMT values and other clinical variables.

**Results:**

The sdLDL-C level was significantly higher in males than in females (*p* <0.05) and there was an age effect on sdLDL-C (*p* <0.05). When the effects of age, gender and other traditional CVD risk factors were adjusted using multiple regression analysis. CA-IMT remained significantly associated with sdLDL-C(*β* = 0.437, *p* <0.001).

**Conclusions:**

There are gender and age differences in sdLDL-C levels among a healthy Chinese population. Moreover, we found adjusted traditional CVD risk factors such as higher age, male sex, and other traditional CVD risk factors, the association between CA-IMT and SdLDL-C remained significant. sdLDL-C is may be a useful predictor in the assessment of CA-IMT in Chinese population.

## Background

Low-density lipoprotein (LDL) consists of a continuum of particles varying in size, density, electrical charge, and chemical (lipid and apo-protein) composition. LDL was identified in the density range 1.019–1.060 g/ml, namely large buoyant LDL (lbLDL), and range 1.034–1.044 g/ml, namely small dense LDL (sdLDL) [1, 2]. Compared with lbLDL, small dense LDL cholesterol (**s**dLDL-C) is thought to be more atherogenic [3]. Indeed, higher levels of sdLDL particles are considered to be more atherogenic compared with lbLDL cholesterol (lbLDL-C) particles; therefore, sdLDL-C is considered to be an important and independent predictor of cardiovascular diseases (CVD) [4–8]. Thus, the selective measurement of sdLDL-C concentrations is useful for evaluating the actual atherogenic risk of individuals. The sdLDL is traditionally measured by ultracentrifugation [9] or gradient gel electrophoresis (GGE) [10] to evaluate the density or LDL particle size, respectively. However, both methods are laborious and require special equipment and a long running time. Nuclear magnetic resonance (NMR) imaging is capable of simultaneously determining the size and number of LDL particles [11]. However, the instrumentation required for NMR is too expensive for general clinical laboratories to acquire feasibly. High performance liquid chromatography (HPLC) enables the determination of lipid concentrations in various lipoprotein subfractions [12]. However, HPLC is also laborious, time-consuming, and expensive for routine clinical use. Hirano et al. [13] have developed a method that uses heparin sodium salt precipitation followed by centrifugation for measuring sdLDL-C, and it still required offline sample pretreatment that hindered its smooth integration into general clinical use. Recently, a new fully automated homogenous assay for measuring sdLDL-C was being used, and sdLDL-C values showed excellent agreement with the sdLDL isolation by sequential ultracentrifugation [14]. This precise and rapid method allows for the routine of large number of samples.

Carotid artery intima-media thickness (CA-IMT) has been utilized as one of the surrogate markers for cardiovascular disease in different populations [15, 16]. CA-IMT increases in high-risk populations such as elderly people [17], those with hypertension [18], diabetes mellitus [19], and chronic kidney disease [20, 21]. Although several studies have reported an association between CA-IMT and LDL particle size distribution in the Japanese and U.S. populations [22–25], it has not been studied in the Chinese population, especially the healthy Chinese population. Furthermore, the measurement of sdLDL-C concentration was useful in the assessment of the presence of CVD [13, 26] and its severity [24]. In addition, a recent publication by Arai et al. [27] demonstrated the utility of the sdLDL-C concentration as a predictive marker for CVD incidence. Since the etiology of CVD is complex, involving environmental and genetic factors that might change from one population to the other, it is important to assess this association in Chinese populations. sdLDL-C is may b a useful predictor in the assessment of CA-IMT in Chinese subjects.

The purpose of this study is to evaluate whether the quantitative measurement of SdLDL-C gives better information in CVD risk assessment than standard LDL-C and other lipid variables using CA-IMT as a surrogate measure of atherosclerosis.

## Results

### sdLDL-C and various parameters in the study subjects

One hundred eighty-three subjects were classified into males group and females group. The age of the subjects ranged from 20 to 78 years old, and the mean age was 45.8 ± 14.6y. Each subject had a normal chemistry and physical profile, and the subject characteristics are summarized in (Table 1). We found that the males group had significantly higher BMI, Systolic BP, TC, sdLDL-C levels than females group (*p* <0.05), and had significantly lower HDL-C level than females group (*p* <0.05).

**Table 1:** Subject characteristics

Variable	Male	Female	*P*
n	95	88	–
Age (year)	47.31 ± 14.58	44.20 ± 14.62	0.153
BMI (kg/m^2^)	23.65 ± 3.61	22.64 ± 3.32	0.042
Systolic BP (mm Hg)	117.37 ± 12.23	113.15 ± 10.80	0.015
Diastolic BP (mm Hg)	83.23 ± 6.34	81.81 ± 5.26	0.088
Family CVD events	43/95	39/88	0.898
FBG (mg/dl)	88.41 ± 7.89	88.87 ± 8.12	0.701
HbA1c (%)	5.34 ± 0.93	5.18 ± 0.87	0.232
TG (mg/dl)	88.83 ± 28.91	82.86 ± 27.89	0.157
TC (mg/dl)	167.94 ± 18.66	161.38 ± 18.55	0.018
HDL-C (mg/dl)	48.43 ± 9.61	54.81 ± 9.64	<0.001
Non- HDL-C (mg/dl)	112.96 ± 15.16	113.13 ± 15.12	0.939
LDL-C (mg/dl)	96.71 ± 12.14	96.72 ± 12.25	0.994
LbLDL-C (mg/dl)	83.23 ± 11.93	82.92 ± 11.69	0.858
SdLDL-C (mg/dl)	14.1 ± 3.9	13.2 ± 3.1	0.041
CA-IMT (mm)	0.54 ± 0.13	0.52 ± 0.14	0.154

Continuous and categorical variables data are expressed as the mean ± standard deviations and real number of subjects, respectively. Means were compared between male and female groups by Student’s t-test. The χ-square test was employed to compare Family CVD events distribution

*Abbreviation*: *BMI* body mass index, *BP* blood pressure, *FBG* fasting blood glucose, *HbAlc* hemoglobin A1c, *TG* triglyceride, *TC* total cholesterol, *HDL-C* high-density lipoprotein cholesterol, *LDL-C* low-density lipoprotein cholesterol, *lbLDL-C* large buoyant LDL-C, *SdLDL-C* small dense LDL-C, *CA-IMT* carotid artery intima-media thickness LbLDL-C (mg/dl) = LDL-C (mg/dl) − SdLDL-C (mg/dl)

### sdLDL-C in the healthy subjects

**Fig. 1:**
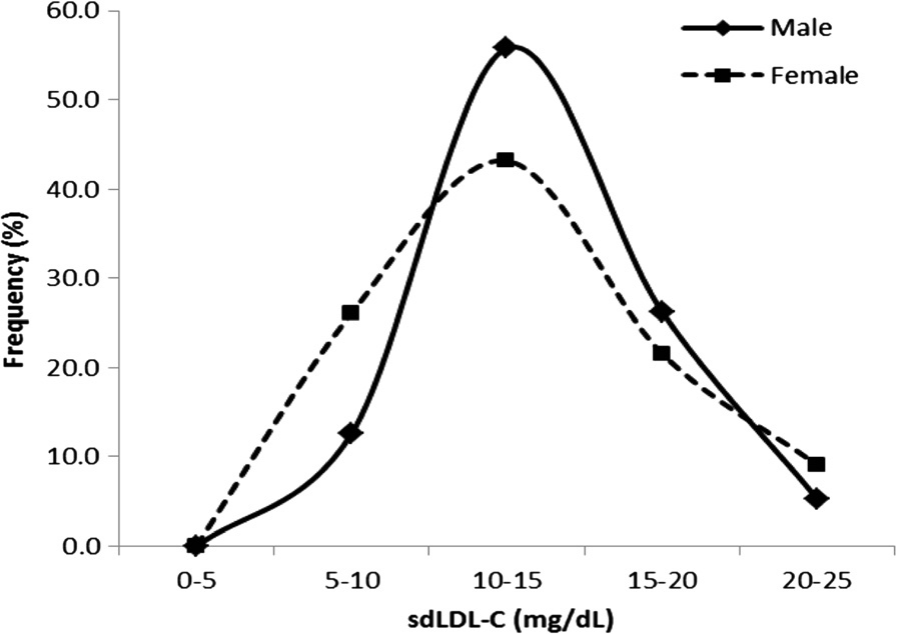
The frequency distribution of small dense low-density lipoprotein cholesterol (sdLDL-C) of healthy males and females with normolipidemia

**Fig. 2:**
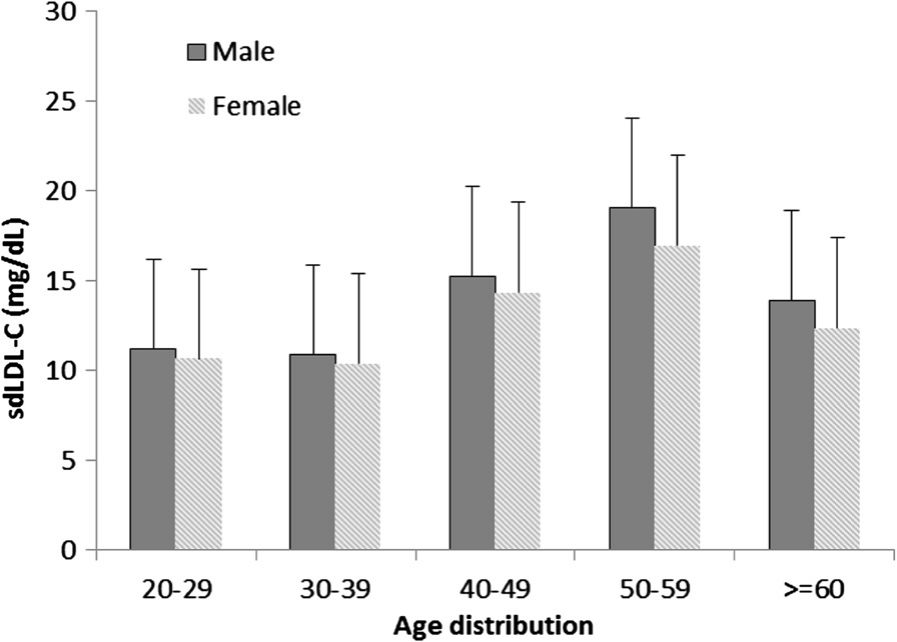
Normal small dense low-density lipoprotein cholesterol (sdLDL-C) concentrations in various age groups and in males and female

We compared the frequency distribution of sdLDL-C among females and males first (Fig. 1) and then separated the healthy female and male population into five age groups (Fig. 2) in order to determine whether there was an age or a gender difference. We found that there was an age effect on sdLDL-C in both males and females. The sdLDL-C level was significantly higher in males above 50 years old than those below 50 years old (16.4 ± 4.1 vs 13.3 ± 3.2 mg/dl, *p* <0.01). Females above 40 years old had a higher sdLDL-C level than those below 40 years old (14.4 ± 3.9 vs 12.1 ± 3.0 mg/dl, *p* <0.01). The percentiles of sdLDL-C value are summarized in (Table 2).

**Table 2:** The percentiles of sdLDL-C value

Variable	sdLDL-C (mg/dl)			
	P_25_	P_50_	P_75_	P_95_
All subjects	10.3	12.5	15.7	21.0
Males	10.6	12.9	16.1	21.6
Females	9.3	12.3	15.6	20.6

Abbreviations are the same as those for Table 1

### Correlation between sdLDL-C and CA-IMT

The results of the Pearson's correlation coefficient analyses revealed that CA-IMT was positively correlated with age (*r* = 0.770, *p* <0.001), systolic BP (*r* = 0.398, *p* <0.001), diastolic BP (*r* = 0.343, *p* <0.001), fasting plasma glucose (*r* = 0.261, *p* <0.001), HbA1c (*r* = 0.192, *p* = 0.009), total cholesterol (*r* = 0.202, *p* =0.006), triglycerides (*r* = 0.226, *p* = 0.002), LDL cholesterol (*r* = 0.340, *p* <0.001), non-HDL cholesterol (*r* = 0.149, *p* = 0.044), large buoyant LDL cholesterol (*r* = 0.182, *p* = 0.014), and sdLDL-C (*r* = 0.436, *p* <0.001), but had no significant association with other clinical variables (Table 3). The results of the multiple regression analysis: First: sdLDL-C as an independent variable adjusted for age and sex revealed that dependent CA-IMT determinants remained significantly associated with sdLDL-C; Second: adjustment for Family CVD history, smoking, systolic BP, diastolic BP, FBG, HbA1c, TC, HDL-C, TC, LDL-C, non-HDL cholesterol, large buoyant LDL cholesterol, and sdLDL-C revealed that dependent CA-IMT determinants remained significantly associated with sdLDL-C (Table 4).

**Table 3:** Correlation between CA-IMT values and sdLDL-C values and other variables

Variable	r	*P* value
Age	0.770	<0.001
BMI	0.106	0.153
Systolic BP	0.398	<0.001
Diastolic BP	0.343	<0.001
FBG	0.261	<0.001
HbA1c	0.192	0.009
TC	0.202	0.006
HDL-C	−0.065	0.385
TG	0.226	0.002
LDL-C	0.340	<0.001
Non-HDL-C	0.149	0.044
LbLDL-C	0.182	0.014
SdLDL-C	0.436	<0.001

Pearson's correlation coefficient analyses were used to explore the relationships

Abbreviations are the same as those for Table 1

**Table 4:** Multivariate associations of CA-IMT with SdLDL-C and other parameters adjusted by age and sex

Variable	Model 1		Model 2	
	*β*	*P*	*β*	*P*
Age	0.562	<0.001	0.422	<0.001
Sex (Male vs. Female)	−0.116	0.269	−0.132	0.067
Family CVD history (Yes vs No)			0.313	<0.001
BMI			0.105	0.155
Smoking (Smokers vs non-smokers)			0.156	0.021
Systolic BP (mm Hg)			0.298	<0.001
Diastolic BP (mm Hg)			0.287	<0.001
FBG (mg/dl)			0.098	0.252
Log TC (mg/dl)			0.052	0.478
HDL-C (mg/dl)			0.061	0.450
LDL-C (mg/dl)			0.123	0.084
SdLDL-C (mg/dl)	0.406	<0.001	0.437	<0.001

Abbreviations are the same as those for Table 1

## Discussion

In this study, we observed that there are gender and age differences in sdLDL-C levels among our tested population, consistenting with the data obtained in the Western populations [26, 28]. Although we have not separated menopausal and premenopausal women, women above 40 years old had a higher sdLDL-C concentration than those below 40 years old. Several studies have consistently shown more favorable lipoprotein profiles among premenopausal women than among men due to estrogen-related protective mechanisms [29, 30]. It is well known that CVD risk markedly increases with aging in men and menopausal women, and alterations in LDL clearly contribute to this increased risk [31].

Moreover, we compared the associations of CA-IMT with SdLDL-C, LDL-C and other various risk factors for CVD in 183 healthy Chinese population. In univariate analysis, CA-IMT was most closely associated with SdLDL-C among the variables tested. This was also true when the effects of age, gender and other traditional CVD risk factors were adjusted using multiple regression analysis. These results suggest that SdLDL-C is a quantitative risk marker of CVD that is more closely associated with CA-IMT than the standard lipid parameters in healthy Chinese population.

Previous studies [22, 24, 25] showed that an association between CA-IMT and sdLDL-C concentrations measured by other methods. Using GGE, Skoglund-Andersson et al. [22] found that plasma concentration of the predominant sdLDL-C subfraction correlated strongly with common CA-IMT in healthy 50-year-old men. Shoji et al. [24] reported that CA-IMT was strongly correlated with sdLDL-C in subjects with dyslipidemia, diabetes mellitus, hypertension, and chronic kidney disease, as well as in smokers by simple precipitation methods. Using NMR, Maeda et al. [25] showed that, compared with TC and LDL-C, abnormalities in HDL-C and sdLDL-C were more strongly and consistently associated with common CA-IMT in patient with moderate chronic kidney disease. In the present study we measured CA-IMT as one of the surrogate markers of atherosclerosis, and found that CA-IMT was more closely associated with SdLDL-C than other lipid parameters tested in healthy Chinese population.

The key finding of this study was that SdLDL-C was a better lipid variable than other standard parameters in assessing the risk of CVD using CA-IMT in healthy population. There are several explanations for this finding. First, SdLDL-C was associated with CA-IMT due simply to its increased level in other traditional CVD risk factors. In fact, we found adjusted traditional CVD risk factors such as higher age, male sex, smoking and Family CVD history, the association between CA-IMT and SdLDL-C remained significant. Therefore, although SdLDL-C is interrelated with other coronary risk factors, such relations do not fully explain the superior association of SdLDL-C with CA-IMT. Second, sdLDL is higher susceptibility to oxidation, and oxidized LDL induces smooth muscle cell proliferation [32], therefore, sdLDL are more atherogenic [33]. sdLDL may initiate the development of carotid atherosclerosis, and we speculate that sdLDL-C concentrations may have a close relationship with CA-IMT.

The present study has some limitations. First, the cohort was relatively small. Second, only the healthy subjects enrolled in the present study. Subjects with coronary risk factors or CVD are required. Finally, being a cross-sectional study, it is not possible to determine a cause and effect relationship between sdLDL-C concentrations and CA-IMT. Further studies are needed to clarify whether CA-IMT is with sdLDL-C concentrations, as measured by homogenous assay, in subjects with coronary risk factors or CVD.

## Conclusion

We observed that there are gender and age differences in sdLDL-C levels among a healthy Chinese population. Moreover, we found adjusted traditional CVD risk factors such as higher age, male sex, smoking and Family CVD history, the association between CA-IMT and SdLDL-C remained significant. sdLDL-C is may be a useful predictor in the assessment of CA-IMT in Chinese population.

## Materials and methods

### Study participants

One hundred eighty-three healthy subjects were randomly collected from consecutive subjects visiting The First People's Hospital of Wujiang, for an annual health checkup from April 2014 to April 2015. The including criteria were: 1) All subjects were native Chinese, and required to have lived in Suzhou since birth. 2) The age should be more than 20 years old. 3) We excluded all subjects with a history of CVD, thromboembolic disease or congestive heart failure; peripheral arterial disease; malignancy; infectious disease; liver or renal disease; overt endocrine disease. In addition, subjects with hypertension, dyslipidemia, diabetes mellitus, as defined by the diagnostic criteria [31, 34, 35] were not included. None of the healthy subjects were currently taking any medications that could influence glycemic and lipid.

All subjects were divided into female group, male group and 5 age groups (20–29, 30–39, 40–49, 50-59, and ≥60 years old). Comparisons of mean values of CA-IMT, BP, sdLDL-C, glucose metabolism and lipid between both males and females, and sdLDL-C concentrations in various age groups and in males and females by Student’s *t*-test. The height and weight of subjects were measured and the body mass index (BMI, weight in kilograms divided by height in meters squared) was calculated. BP was measured in the morning (after a 12 h fast) by the same investigator with a sphygmomanometer on the right arm of the subject after a 10-min rest in the supine position. We performed physical examinations and CA-IMT examinations in the morning (after a 12 h fast) and obtained blood samples from the antecubital vein for serum and plasma analyses at the same time. This study was approved by the local ethics committee and was carried out according to the principles of the Declaration of Helsinki. Each subject provided informed consent.

### Carotid ultrasonography

A trained technicians who was blinded to the clinical characteristics of subject groups evaluated the wall thickness of the carotid arteries in all participants using high-resolution B-mode ultrasound (Philips iU22 ultrasound System) equipped with a L9-3 (7.5 MHz) linear array transducer (Philips Healthcare, Best, The Netherlands) [36]. After the subject had rested for at least 10 min in the supine position with the neck in slight hyperextension, we evaluated the optimal visualization of the common carotid arteries, carotid bulb, and extracranial internal and external carotid arteries bilaterally. CA-IMT was assessed as the greatest CA-IMT at any location in the far walls of these carotid arteries on both sides. The max CA-IMT was defined as the greater of the 2 unilateral CA-IMT values [37].

### Laboratory measurements

Fasting blood glucose (FBG), Total cholesterol (TC), Triglyceride (TG), High-density lipoprotein cholesterol (HDL-C), and LDL cholesterol (LDL-C) were measured by standard laboratory procedures. An automatic biochemistry analyzer (Hitachi-7600; Hitachi, Tokyo, Japan) was used for all above tests, using standard kits. Hemoglobin A1c (HbA1c) level was determined by HPLC, using automatic analyzer (ADAMS A1c HA-8181, Arkray). The value of HbA1c (%) was estimated as a National Glycohemoglobin Standardization Program (NGSP) equivalent value (%) derived from the Japanese Diabetes Society (JDS) value and calculated by the formula HbA1c (%) = HbA1c (JDS) (%) +0.4 % [38]. The sdLDL-C was measured using a newly developed homogenous assay in an automatic analyzer (Hitachi-7600; Hitachi, Tokyo, Japan), and commercial kit (sdLDL-C, SEIKEN; Denka Seiken, Tokyo, Japan) was used for determination as we previously reported [39].

### Statistical analysis

Continuous data are presented as mean ± SD, and comparisons by Student’s *t*-test. The χ-square test was employed to compare categorical data. The difference in frequency data was determined by Chi-squared test. Pearson's correlation coefficient analyses were used to explore the relationships between CA-IMT values and sdLDL-C values and other clinical variables. Multiple regression analysis was performed using CA-IMT values, sdLDL-C values, and those of the other clinical variables. All the probability values were 2-tailed and differences with *P* <0.05 were considered to be statistically significant. All tests were performed by SPSS program version 20.0.

## Abbreviations

BMI: body mass index
BP: blood pressure
CA-IMT: carotid artery intima-media thickness
CVD: cardiovascular diseases
GGE: gradient gel electrophoresis
HbAlc: hemoglobin A1c
HDL: high-density lipoprotein
HDL-C: HDL cholesterol
HPLC: high performance liquid chromatography
lbLDL: large buoyant LDL
LDL: low-density lipoprotein
LDL-C: LDL cholesterol
NMR: nuclear magnetic resonance
sdLDL: small dense LDL
sdLDL-C: small dense low-density lipoprotein cholesterol
TC: total cholesterol
TG: triglyceride
